# Tau Aggregates in Cerebrospinal Fluid: A Promising Biomarker for Neurodegenerative Diseases

**DOI:** 10.1002/alz.086773

**Published:** 2025-01-09

**Authors:** Md Tohidul Islam, Thomas K Karikari, Kaj Blennow

**Affiliations:** ^1^ Göteborg Universitet, Gothenburg, Västra Gotland Sweden; ^2^ Institute of Neuroscience and Physiology, Department of Psychiatry and Neurochemistry, The Sahlgrenska Academy at University of Gothenburg, Mölndal, PA Sweden; ^3^ Department of Psychiatry, School of Medicine, University of Pittsburgh, Pittsburgh, PA USA; ^4^ Clinical Neurochemistry Laboratory Sahlgrenska University Hospital, Mölndal Sweden; ^5^ Institute of Neuroscience and Physiology, Department of Psychiatry and Neurochemistry, The Sahlgrenska Academy, University of Gothenburg, Mölndal Sweden

## Abstract

**Background:**

This research introduces a novel method for quantifying aggregated tau in body fluids, specifically cerebrospinal fluid (CSF), aiming to enhance the diagnosis and monitoring of neurodegenerative diseases, with a focus on Alzheimer's disease (AD).

**Method:**

By combining tau protein amplification with a highly sensitive single‐molecule array (Simoa) immunoassay using an anti‐tau antibody CT19.1 in a homogenous manner, the approach enables precise measurements of tau aggregates in CSF.

**Result:**

Preliminary testing involved 40 CSF samples from AD and non‐AD individuals, treated with a recombinant tau amplifier that we innovated in our research facility and subjected to an extended incubation period. Without amplification, the immunoassay showed baseline signals. However, after amplification, the technique not only elevated signals to a detectable range but also differentiated AD CSF from non‐AD samples.

**Conclusion:**

Our methodology integrates tau amplification with a remarkably sensitive immunoassay detection system, allowing for precise quantification of tau aggregates in CSF. The approach is to adapt into the assay for plasma and serum samples. This expansion of the method's applicability has the potential to revolutionize non‐invasive diagnostic tools for neurodegenerative diseases. This innovation opens a possibility to facilitate the tracking of disease progression and the assessment of treatment responses, thereby making significant contributions to the advancement of clinical research and therapeutic trials. Another important aspect of this approach is the use of the assay antibody CT19.1 which is generated in our facility. This antibody binds to the pathologically significant MTBR region, which is considered the core region that facilitates tau aggregation, which indicates a possibility to explore further as therapeutic intervention.

